# Retraction Note: Knockdown long non-coding RNA ANRIL inhibits proliferation, migration and invasion of HepG2 cells by down-regulation of miR-191

**DOI:** 10.1186/s12885-024-12033-y

**Published:** 2024-03-04

**Authors:** Deyu Huang, Chunhua Bi, Qingxi Zhao, Xueli Ding, Cheng Bian, Hui Wang, Ting Wang, Hua Liu

**Affiliations:** 1 Department of Infectious Disease, The Affiliated Hospital of Qingdao University, 266003 Qingdao, Shandong China; 2 Department of Gastroenterology, The Affiliated Hospital of Qingdao University, No.16, Jiangsu Road, 266003 Qingdao, Shandong China

**Retraction Note**: ***BMC Cancer *18, 919 (2018)**

10.1186/s12885-018-4831-6

The Editors have retracted this article because of significant concerns regarding several Figures presented in this work, which question the integrity of the data. After publication, overlap was detected between Figs. 2A and 4E of [1]. In addition, an investigation by the Chinese Ministry of Science and Technology found the contents of the paper to be unreliable.

The Editors therefore no longer have confidence in the integrity of the data in this article.

None of the authors have responded to any correspondence from the editor/publisher about this retraction.
